# Effects of melatonin on the proliferation and differentiation of rat adipose-derived stem cells

**DOI:** 10.4103/0970-0358.41104

**Published:** 2008

**Authors:** Arash Zaminy, Iraj Ragerdi Kashani, Mohammad Barbarestani, Azim Hedayatpour, Reza Mahmoudi, Safoura Vardasbi, Mohammad Ali Shokrgozar

**Affiliations:** Department of Anatomy, School of Medicine, Medical Sciences, University of Tehran, Tehran, Iran; 1 Department of Anatomy, School of Medicine, Yasouj University of Medical Sciences, Tehran, Iran; 2 Department of Biochemistry, School of Medicine, Medical sciences, University of Tehran, Tehran, Iran; 3 National Cell Bank of Iran, Pasteur Institute of Iran, Tehran, Iran

**Keywords:** Adipose tissue, differentiation, melatonin, osteogenic, stem cells

## Abstract

**Background:**

Osteogenesis driven by adipose-derived stem cells (ADSCs) is regulated by physiological and pathological factors. Accumulating evidence from *in vitro* and *in vivo* experiments suggests that melatonin may have an influence on bone formation. However, little is known about the effects of melatonin on osteogenesis, which thus remains to be elucidated. This study was performed to determine whether melatonin at physiological concentrations (0.01-10 nM) could affect the *in vitro* proliferation and osteogenic differentiation of rat ADSCs.

**Materials and Methods:**

ADSCs were isolated from the fat of adult rats. After cell expansion in culture media and through three passages, osteogenesis was induced in a monolayer culture using osteogenic medium with or without melatonin at physiological concentrations (0.01-10 nM). After four weeks, the cultures were examined for mineralization by Alizarin Red S and von Kossa staining and for alkaline phosphatase (ALP) activity using an ALP kit. Cell viability and apoptosis were also assayed by 3-(4, 5-dimethylthiazol-2-yl)-5-(3-carboxymethoxyphenyl)-2-(4-sulfophenyl)-2H-tetrazolium (MTT) assay and flow cytometry, respectively.

**Results:**

The results indicated that at physiological concentrations, melatonin suppressed proliferation and differentiation of ADSCs. These data indicate that ADSCs exposed to melatonin, had a lower ALP activity in contrast to the cells exposed to osteogenic medium alone. Similarly, mineral deposition (calcium level) also decreased in the presence of melatonin. Flow cytometry confirmed that cell growth had decreased and that the numbers of apoptotic cells had increased.

**Conclusion:**

These results suggest that the physiological concentration of melatonin has a negative effect on ADSC osteogenesis.

## INTRODUCTION

The repair of bone defects secondary to trauma, osteomyelitis, fracture nonunion and tumor resection, poses a significant problem for many clinicians, particularly, plastic, head and neck and orthopedic surgeons.[1]

Mesenchymal stem cells have recently received widespread attention because of their potential use in tissue engineering applications.[2] Mesenchymal stem cells (MSCs) are defined as self-renewable, multipotent progenitor cells with the capacity to differentiate into several distinct mesenchymal lineages.[3]

Bone marrow provides the most universal source of MSCs and the apparent pluripotent nature of bone marrow stem cells (BMSCs) makes them excellent candidates for tissue engineering. However, BMSCs have been reported to require selective lots of sera and growth factor supplements for culture expansion.[4] Moreover, traditional bone marrow procurements, especially in volumes larger than a few milliliters may be painful, frequently requiring general or spinal anesthesia[4–6] and may yield low numbers of MSCs upon processing.[7] As an alternative to BMSCs, adipose tissue is particularly attractive because of its easy accessibility and abundance.[8–10] Adipose-derived stem cells (ADSCs) obtained from lipoaspirates have been shown to have the multilineage potential to differentiate into adipogenic, chondrogenic, myogenic and osteogenic cells.[9,11]

ADSCs mineralize their extracellular matrix (ECM) and show increased expression of osteocalcin and alkaline phosphatase (ALP).[12] These factors may make ADSCs a viable clinical alternative to BMSCs.

Melatonin has been shown to play a role in many physiological systems including those involved in sleep, gastrointestinal physiology, immune defence, cardiovascular function, detoxification, reproduction and bone physiology.[13]

Melatonin influences cell proliferation and the effect of stimulation or suppression of cell division appears to depend on its concentration and the cell type examined.[14] Melatonin's ability to directly promote osteoblast maturation was first demonstrated in preosteoblast and rat osteoblast-like osteosarcoma cells. In these cells, low concentrations of melatonin increased the mRNA levels of several genes expressed in osteoblasts including bone sialoprotein (BSP), alkaline phosphatase (ALP), osteopontin and osteocalcin.[15] Several studies using various animal models show that melatonin prevents bone deterioration including preventing idiopathic scoliosis in adolescents[13,16] and that it stimulates proliferation of normal cells such as human bone cells.[13] However, there are no reports of melatonin effects on ADSC osteogenic differentiation. In this study, we examined the effects of low and high (0.01-10 nM) physiological concentrations of melatonin on the proliferation, apoptosis and differentiation of ADSCs derived from adult rat using *in vitro* culture systems.

## MATERIALS AND METHODS

### Isolation of adipose-derived stem cells

About six to eight - weeks’ old male rats were sacrified using Diethyl ether. The groin and testicular areas were shaved and prepared with standard sterile techniques. A 15 mm incision was made along the scrotum and the testes were pulled outward, exposing the epididymal fat pad. Epididymal adipose tissue was excised, placed on a sterile glass surface and finely minced. The tissue was then transferred to a 50 ml conical tube and washed in Hank's Balanced Salt Solution (HBSS, Sigma). The minced tissue was placed in a sterile, 50 ml conical tube (Greiner, Germany) containing 0.05% tissue culture grade collagenase type 1 (Sigma, St. Louis, MO.) and 5% bovine serum albumin (BSA, Sigma). The tube was incubated at 37°C for 1 h and shaken every five minutes. Next, an equal volume of Dulbecco's Modified Eagle Medium (DMEM) with 10% fetal bovine serum (FBS) was added to neutralize the collagenase. The tube contents were filtered through a sterile 250 *μ*m nylon mesh to remove undigested debris. The digested tissue was then centrifuged at 250 *g* for five minutes and mature adipocytes were removed by aspirating the supernatant, leaving behind a pellet of cells. The cell pellet was resuspended in adipose-derived stem cell medium: DMEM/F12 (Sigma.), 10% FBS (Gibco), 100 U/ml penicillin and 100 *μ*g/ml streptomycin (Sigma). Cell counts were determined with a haemocytometer.[2]

*Cell culture and expansion:* The collected fat-derived stem cells were plated in 75 cm^2^ vented tissue culture flasks at a density of 1 × 10^6^ cells per flask in DMEM with 10% FBS and 1% penicillin/streptomycin (Sigma, USA). The flasks were maintained in a tissue culture incubator at 37°C in an atmosphere containing 5% carbon dioxide. The media were replaced the day after the initial stem cell harvest and every third day after that. The cells were monitored for confluence on a daily basis.

The cells were subcultured when the flasks reached 80% confluence. The media were removed and cells detached by incubation with 3 ml trypsin for 5 minutes. The cell layer was then collected into a 15 ml conical tube and was centrifuged at 250 *g* for ten minutes. The pellet was resuspended with 10 ml control media and the cells were counted. The cells were seeded into other flasks at a density of 10 × 10^5^ cells per flask.

ADSCs were cultured and expanded in basal medium and used for experiments at passage 3.

*Osteogenic differentiation:* After culture expansion to three passages, the cells were trypsinized and replated in T75 tissue culture flasks at a density of 10 × 10^5^ cells per flask Cells were allowed to adhere and grow for three days and the media replaced with osteogenic media consisting of DMEM with 10% fetal bovine serum (Sigma, USA), 0.1 *μ*M dexamethasone (Sigma, USA), 10 mM beta-glycerol phosphate (Sigma, USA), 50 *μ*g/ml ascorbic acid-2-phosphate (Sigma, USA) with or without physiological concentrations of melatonin (0.01-10 nM) (Sigma, USA).[2]

*Treatment groups:* Three treatment groups: osteogenic medium alone, osteogenic medium with low physiological concentration of melatonin (0.01 nM) and osteogenic medium with a high physiological concentration of melatonin (10 nM), were used throughout this study to analyze melatonin's effect on ADSC differentiation into osteoblasts. These studies were conducted for 28 days to determine if melatonin could modulate osteogenic differentiation of ADSCs into osteoblasts.

*Confirmation of osteogenic differentiation:* Confirmation of osteogenesis was done by means of von Kossa and Alizarin Red S staining (to highlight extracellular matrix calcification) and the assessment of alkaline phosphatase activity.

*Von kossa staining:* The cells in flasks (25 cm^2^) were rinsed with phosphate-buffered saline (PBS) (Sigma, USA) and fixed in 4% paraformaldehyde (Sigma, USA) for 20 min. The cells were incubated in 5% silver nitrate (Gibco, USA) in the dark and the flasks were exposed to ultraviolet light for 1 h. The secretion of calcified extracellular matrix was observed as black nodules with von Kossa staining.[12]

*Alizarin red s (ARS) staining:* The cells in T25 flasks were washed with PBS and fixed in 10% (v/v) formaldehyde (Sigma, USA) at room temperature for 20 min. The monolayer was washed twice with excess distilled water prior to the addition of ARS 2% (Sigma, USA) (pH 4.1) per flask. The flasks were incubated at room temperature for 20 min while being shaken. After the aspiration of the unincorporated dye, the flasks were washed four times with distilled water while being shaken for 5 min.[17]

*Quantification of mineralization:* The analysis of the amount of calcium deposition in osteogenic medium was modified from a previous report. For the quantification of Alizarin Red S staining, 2 ml 10% (v/v) acetic acid was added to each well and the plate was incubated at room temperature for 30 min while being shaken. The monolayer was scraped off the plate with a cell scraper and transferred to a 15 ml micro centrifuge tube with a wide-mouth pipette after the addition of 1ml 10% (v/v) acetic acid to the scraped cells. After vortexing for 30 s, the slurry was overlaid with 1.25 ml mineral oil (Sigma, USA), heated to exactly 85°C for 10 min and transferred to ice for 5 min. The slurry was then centrifuged at 20,000 *g* for 15 min and 500 *μ*l of the supernatant was removed to a new 1.5 ml micro centrifuge tube. Two hundred microliters of 10% (v/v) ammonium hydroxide was added to neutralize the acid. In some cases, the pH was measured at this point to ensure that it was between 4.1 and 4.5. Absorbance of aliquots (150 *μ*l) of the supernatant was measured in triplicate at 405 nm in 96 well format using opaque-walled, transparent-bottomed plates.[17]

*ALP activity:* Cells were lysed by sonication for three cycles and centrifuged at 2000 *g* for 15 min at 4°C. The supernatant was kept at −20°C for the analysis of ALP activity and protein content. Protein samples were analyzed for total cellular protein concentrations and ALP activity. The total protein content of each sample was determined according to Bradford's method, using bovine serum albumin (BSA) as a standard. The ALP activity was performed using an ALP kit (Ziest Chem, Tehran, Iran) according to the manufacturer's instructions. The levels of activity were normalized with the amounts of protein in the cell lysates (units/mg protein).[18]

*Cell viability assay:* The MTT [3-(4, 5-dimethylthiazol-2-yl)-2, 5-diphenyltetrazolium bromide] (Sigma, USA) test measures the mitochondrial (metabolic) activity in the cell culture, which reflects the number of viable cells. In brief, the cultures (10 × 10^5^ seeded per well in a 96well plate) were washed with PBS and 200 *μ*l MTT reagent added. Following incubation for 3 hrs in the incubator (in 5% CO_2_ at 37°C), the absorption of the medium was measured in an ELISA Reader (Anthon 2020) at 440 nm.[19]

*Apoptosis detection:* DNA fragmentation, as a late feature of apoptosis, was determined by flow cytometry as a percentage of nuclei with hypodiploid DNA content. For DNA content evaluation, the samples were fixed with 70% ethanol at 4°C for at least 30 min. They were washed in PBS, resuspended in 400 *μ*l citrate buffer (Sigma, USA) and then stained with 400 *μ*l of a 50 *μ*g/ml propidium iodide PI (Sigma, USA). The samples were incubated at 37°C for 30 min and then analyzed. PI red fluorescence was collected on a linear scale: the events in the hypodiploid peak identified the percentage of apoptosis. Sample acquisition was performed by FACScan flow cytometer equipped with Cell Quest software.[20]

### Statistical analysis

The results are listed as the mean ± SE. The statistical difference was analyzed by one-way ANOVA followed by Dunnett's test. *P* < 0.05 was considered to be statistically significant. All assays were performed in triplicate.

## RESULTS

**Figure 1:**
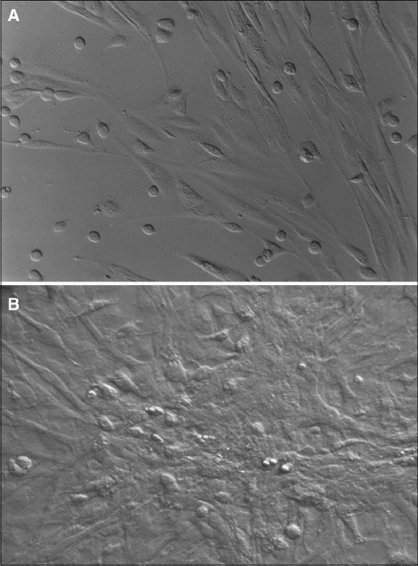
Initially adherent adipocyte stem cells grew as spindle-shaped cells that developed into multipolar fibroblastoid cells (A). They gradually reached confluency on the 10^th^ day

Isolated Rat Adipose-Derived Stem Cells: Adipose-derived stem cells grown in culture appeared spindle-shaped. Cells cultured in osteogenic media demonstrated a dramatic change in morphology from day 10 post induction, with the cells changing morphology from an elongated fibroblastic appearance to a polygonal shape [Figure 1].

**Figure 2:**
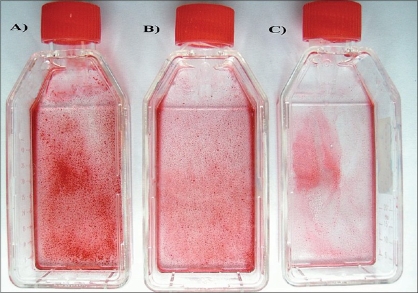
Alizarin Red S staining after osteogenic differentiation. Staining for mineral deposition was performed for ADSCs after 28 days. The osteogenic medium alone group was the control group. (A) Control medium (B) in presence of melatonin 10 nM, (C) in presence of melatonin 0.01 nM

*Effects of melatonin on formation of mineralized bone nodules:* The cytological results of ADSC cultures were convincingly positive when stained by Alizarin Red S and von Kossa after 28 days after subculture. Alizarin red S staining was used to investigate mineralized matrix formation by ADSCs. As shown in Figure 2, the intensity of staining increased in cells cultured in osteogenic medium alone when compared with cells exposed to osteogenic medium supplemented with melatonin [Figure 2].

**Figure 3:**
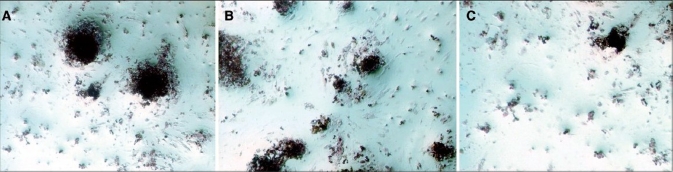
Von Kossa staining after osteogenic differentiation of adipocyte stem cells. This staining was done only for demonstrating osteogenic differentiation after 28 days. (A) Osteogenic medium alone, (B) Osteogenic medium in presence of melatonin10 nM, (C) Osteogenic medium in presence of melatonin 0.01 nM

Nodule formation by ADSCs was investigated with von Kossa staining. The first mineralized bone nodules formed by ADSCs were observed after 10 days of treatment. The addition of melatonin at physiologic concentrations to the osteogenic media decreased the formation of mineralized nodules compared with osteogenic media alone [Figure 3]. The experiments were repeated at least three times and showed similar effects.

**Figure 4:**
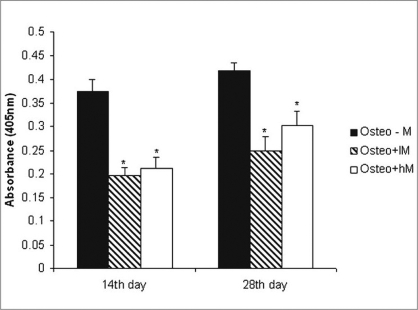
Calcium deposition. The ADSCs were cultured in osteogenic medium in the absence and presence of physiological concentrations of melatonin (0.01-10 nM) for 28 days in flasks (25 cm^2^) as a monolayer culture. The osteogenic medium alone group was the control group. Calcium depositions were measured as described in Materials and Methods. Values are mean ± SE (*P < 0.05). AD: Adipose-derived stem cells, Cont: control, lM: melatonin 0.01 nM, hM: melatonin 10 nM

*Quantification of mineralization:* Calcium level quantification was measured in three groups after 14 and 28 days following differentiation of ADSCs in osteogenic medium with or without of melatonin [Figure 4].

**Figure 5:**
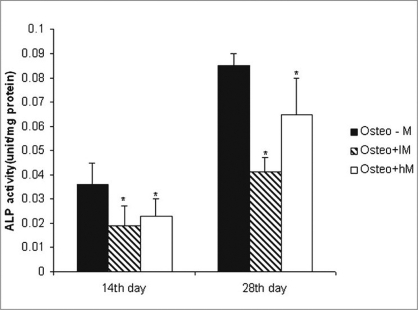
ALP activity in adipocyte stem cells on osteogenic differentiation on days 14 and 28. The cells were plated at 10 × 10^5^ cells/flask and cultured in osteogenic medium with or without physiologic concentration of melatonin. The osteogenic medium alone group was the control group. Values are mean ± SE (*P < 0.05). ADSCs: Adipose-derived stem cells, Cont: control, lM: melatonin 0.01 nM, hM: melatonin 10 nM

*Effects of melatonin on ALP activity:* As a marker for ADSC differentiation into osteoblasts, ALP levels were measured after 14 and 28 days. As shown in Figure 5, ALP activity increased in ADSCs following incubation in osteogenic medium alone when compared with cells exposed to osteogenic medium containing melatonin [Figure 5].

**Figure 6:**
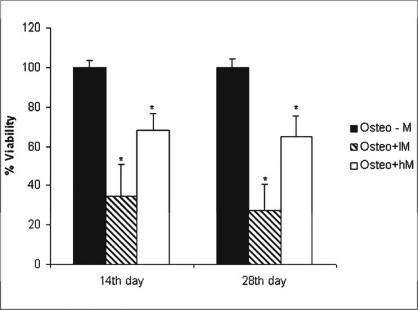
Effect of physiologic concentration of melatonin on the viability of adipose-derived stem cells. The cells were plated at 10 × 10^5^ cells/well and cultured in osteogenic medium with or without physiologic concentration of melatonin for 28 days. Cell viability was measured by MTT assay on 14^th^, 28^th^ days. The osteogenic medium alone group was the control group. Values are mean ± SE (n = 3) (*P < 0.05). Cont: control, lM: melatonin 0.01 nM, hM: melatonin 10 nM

*Effects of melatonin on viability of ADSCs:* The physiological concentrations of melatonin (0.01-10 nM) affect viability of ADSCs at 14 and 28 days after melatonin treatment as assessed by the MTT assay [Figure 6].

### Effects of melatonin on apoptosis of ADSCs

**Figure 7:**
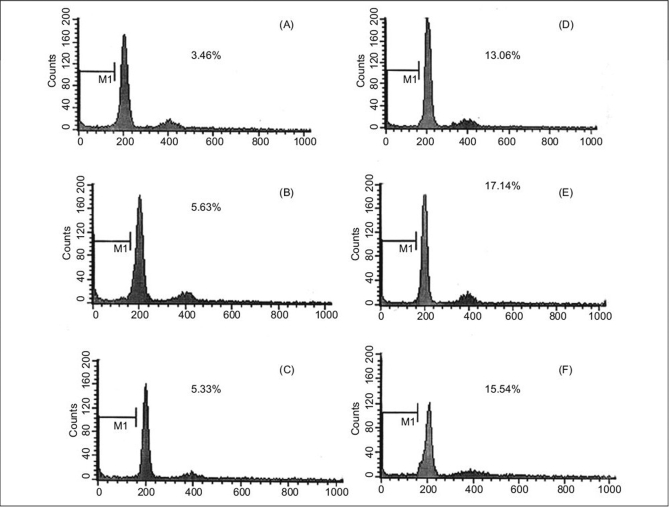
Flow cytometric analysis of DNA content for detection of apoptosis after 24 and 72 h of melatonin treatment. The cells were plated at 10 × 10^5^ cells/flask and cultured in osteogenic medium in presence of melatonin. A, D after 24, 72 h treatment with osteogenic medium alone, respectively. B, E: after 24, 72 h treatment with osteogenic medium in presence of melatonin 0.01 nM, respectively. C, F: after 24, 72 h treatment with osteogenic medium in presence of melatonin 10 nM, respectively

To accurately quantitate the incidence of apoptotic cells in the defined ADSCs population, we used flow cytometry to detect content of DNA in cells labeled by propidium iodide. As shown in Figure 7, the incidence of apoptotic ADSC cells is increased following incubation in osteogenic medium supplemented with melatonin when compared with cells exposed to osteogenic medium alone. A decrease in cell growth was detected after 24 and 72 h of melatonin treatment but growth again increased with prolonged treatment.

## DISCUSSION

Our results clearly show that melatonin modulates the proliferative and differentiative ability of ADSCs in a concentration- and time-dependent manner.

One of the most interesting functions of physiological concentrations of melatonin in the present study was that ADSCs which had been propagated in melatonin-containing media exhibited a decrease in proliferation and osteogenic differentiation. The researchers observed when ADSCs were exposed to melatonin, ALP activity and mineral deposition decreased (13, 14). This decrease can be due to the reduction in the number of cells, which was demonstrated by the results of both the MTT assay and flow cytometry. In the present study, it was demonstrated that in melatonin-containing media, the progression of the apoptotic type of ADSCs is not prevented; rather, it was exaggerated by melatonin. The induction of apoptosis by melatonin in the absence of other drugs is not a common effect of this indole in normal cells. Melatonin influences cell proliferation and differentiation and the effect of stimulation or suppression of cell division appears to depend on its concentration and the cell type examined.[14] Antiproliferative effects of melatonin have been demonstrated *in vivo* and *in vitro* in a number of cancer cells[21,22] and normal cells.[23,24] In contrast, pharmacological concentrations of melatonin (10-100 mM) stimulate the proliferation of normal cells such as human bone cells.[17,25] The fact that melatonin may affect cell growth in a biphasic manner was first reported by Slominski and Pruski in experiments with cultured rat melanoma cells.[26] In that study, low concentrations of melatonin suppressed human melanoma cell growth, while higher concentrations stimulated such growth. These findings led Roth *et al*. to explore the effect of melatonin on cell growth in rat pheochromacytoma cells (PC12 cells).[27]

Similar to the above findings with human melanoma cells, melatonin induced a biphasic dose response for cell growth in the PC12 cell model. More specifically, at low concentrations, melatonin suppressed PC12 cell growth, while at a higher concentrations, it prevented cell death.[27] Thus, melatonin differentially suppressed proliferation in different cell lines. Melatonin's behavior was complex, affecting the cell cycle presumably by different mechanisms according to its dose. The intensity of the different responses to melatonin could be related to the cell - line-specific pattern of melatonin cellular receptors and cytosolic protein expression.[26]

In summary, it is premature to offer firm conclusions about such findings except to indicate that biphasic apoptotic responses reliably occur. However, the fact that these observations are quite recent suggests that they will stimulate more exploration in this important low-dose research area.
